# Global shifts in species richness have shaped carpet shark evolution

**DOI:** 10.1186/s12862-021-01922-6

**Published:** 2021-10-21

**Authors:** Bret M. Boyd, Jason C. Seitz

**Affiliations:** 1 Center for Biological Data Science, Virginia Commonwealth University, Richmond, VA 23284 USA; 2 ANAMAR Environmental Consulting, Inc, 2106 NW 67th Place, Suite 5, Gainesville, FL 32653 USA

**Keywords:** Orectolobiformes, Biodiversity hotspot, Dispersal, Phylogenetics, Fossil

## Abstract

**Background:**

The evolutionary processes that shape patterns of species richness in marine ecosystems are complex and may differ between organismal groups. There has been considerable interest in understanding the evolutionary processes that led to marine species richness being concentrated in specific geographical locations. In this study we focus on the evolutionary history of a group of small-to-medium sized sharks known as carpet sharks. While a few carpet shark species are widespread, the majority of carpet shark species richness is contained within a biodiversity hotspot at the boundary of the Indian and Pacific oceans. We address the significance of this biodiversity hotspot in carpet shark evolution and speciation by leveraging a rich fossil record and molecular phylogenetics to examine the prehistoric distribution of carpet sharks.

**Results:**

We find that carpet sharks species richness was greatest in shallow seas connected to the Atlantic Ocean during the Late Cretaceous, but that there was a subsequent loss of biodiversity in Atlantic waters. Fossil evidence from sites in close geographic proximity to the current center of carpet shark diversity are generally restricted to younger geologic strata.

**Conclusions:**

From this data we conclude that (1) center of carpet shark biodiversity has shifted during the last 100 million years, (2) carpet sharks have repeatedly dispersed to nascent habitat (including to their current center of diversity), and (3) the current center of carpet shark biodiversity conserves lineages that have been extirpated from this prehistoric range and is a source of new carpet shark species. Our findings provide insights into the roles of marine biodiversity hotspots for higher-tropic level predators and the methods applied here can be used for additional studies of shark evolution.

## Background

Geographical regions that contain a rich taxonomic diversity, across multiple taxonomically distant groups, with many endemic species are known as biodiversity hotspots [1]. The assembly of diverse species communities in marine biodiversity hotspots has provided insights into the evolution of marine species and ecosystems [2–4]. The region at the boundary of the Indian and Pacific oceans represents a large marine biodiversity hotspot, housing a rich diversity of shallow-water marine and reef species [2, 4–6]. There has been considerable interest in understanding the evolutionary and biogeographical processes that have shaped species diversity and composition in this region [2–4, 6]. This region appears to have been a cradle of biodiversity, by facilitating speciation during the Neogene [2–4, 6]. However, some of the lineages predate the formation of the biodiversity hotspot at the boundary of the Indian and Pacific oceans, suggesting these lineages immigrated to the region and then speciated [2]. Phylogenetic analysis and fossil evidence suggests that similar marine biodiversity hotspots were present near what is now southwest Europe during the Paleogene and that many of these species-groups present there immigrated into the current center of biodiversity during the Neogene [2]. Much of this work focused on coastal plants or small marine animals with rich fossil records [2]. However, recent studies suggest that marine predators may have followed a similar evolutionary path, diversifying elsewhere and subsequently arriving at the boundary of the Indian and Pacific oceans, where they then radiated [7, 8]. Here we examine the evolutionary history of carpet sharks (Chondrichthyes: Orectolobiformes) to assess their prehistoric distribution and estimate the age of species divergence times to provide insights into the colonization of a biodiversity hotspot by small-to-medium sized marine predators.

Carpet sharks represent a monophyletic group that encompasses a rich diversity of extant [7, 9–12] and fossil species [13, 14]. Extant carpet shark species (45 extant species within 7 families) constitute mostly small-to-medium-sized sharks (< 3.2 m long) and inhabit warm to temperate waters of continental and insular shelves [9, 15]. The only exception, the pelagic whale shark (*Rhincodon typus*), is the largest fish in the world [10, 11, 16]. Most carpet shark species have teeth that can rotate to accommodate either soft or hard-bodied prey [17] and this tooth morphology is unique among sharks [13, 18] (E. Manning pers. comm.). This has allowed many carpet shark species to prey on both small fishes and cephalopods (soft-bodied) as well as invertebrates with hard shells (hard-bodied), taking advantage of a variety of dietary resources [19, 20]. However, some groups of carpet sharks have become dietary specialists. The whale shark is largely planktivorous [19] and has reduced teeth [13, 14, 18]. The wobbegongs (Orectolobidae) and blind sharks (Brachilaridae) are higher-level predators that consume bony fishes, cephalopods, and smaller sharks [21, 22]. The wobbegong’s teeth are modified for a diet of soft-bodied prey, while blind sharks retain the characteristic rotating teeth [17]. The greatest species richness of carpet sharks is centered within the eastern Indian and western Pacific oceans, with a total of 41 species of carpet sharks are present in this region, 39 species that are endemic to one, or both of these oceans [10, 23, 24]. The richness of carpet shark species within a marine biodiversity hotspot and a rich fossil record made carpet sharks ideal to study the role of biodiversity hotspots in the speciation of marine predators.

In order to describe the evolutionary history of carpet sharks, we first needed estimate species divergence times. The process of estimating species divergence times from molecular sequence data requires external information to calibrate the rate at which new substitutions arise and are fixed. Fossils provide the most reliable source of information to calibrate DNA substitution rates and estimate divergence times [25]. The fossilized remains of shark teeth are commonly encountered in paleontological studies [13, 14], because a typical shark loses tens of thousands of teeth during a lifetime [26]. A few studies have used fossil shark evidence described in the primary literature to obtain divergence calibration times [8, 27–32]. The results of these studies suggest the literature contains useful and well-founded information on fossilized shark teeth that can be used to calibrate molecular phylogenies; however, these studies highlight issues around selecting the optimal fossil to calibrate the rate of DNA substitutions among many potentially informative fossils. These studies either selected the oldest fossil within a specious and evolutionarily old clade [27–32] or hand selected a few fossils representing different chronologically distant constraints [32]. The first approach excludes many potentially informative fossils and the later could lead to inconsistencies between studies. Here we employ a simple and repeatable method for quickly screening hundreds of fossil descriptions from the primary literature to find the optimal fossils for calibrating when to species of sharks diverged in geological time, while avoiding the time-consuming re-examination of fossils, and avoiding the assumption that all fossils are correctly identified within the literature. We then used these fossils to build a time-calibrated phylogenetic tree, comparing, in three cases, estimates of species divergence times to expected divergence times to evaluate our approach. Once we had a phylogenetic tree representing both species relationships and species divergence times, we needed to identify prehistoric centers of biodiversity. We did this by classifying to family the hundreds of fossils evaluated for the molecular calibration. From this data we identified regions that contained the greatest family level diversity at different points in the past.

## Results

**Fig. 1:**
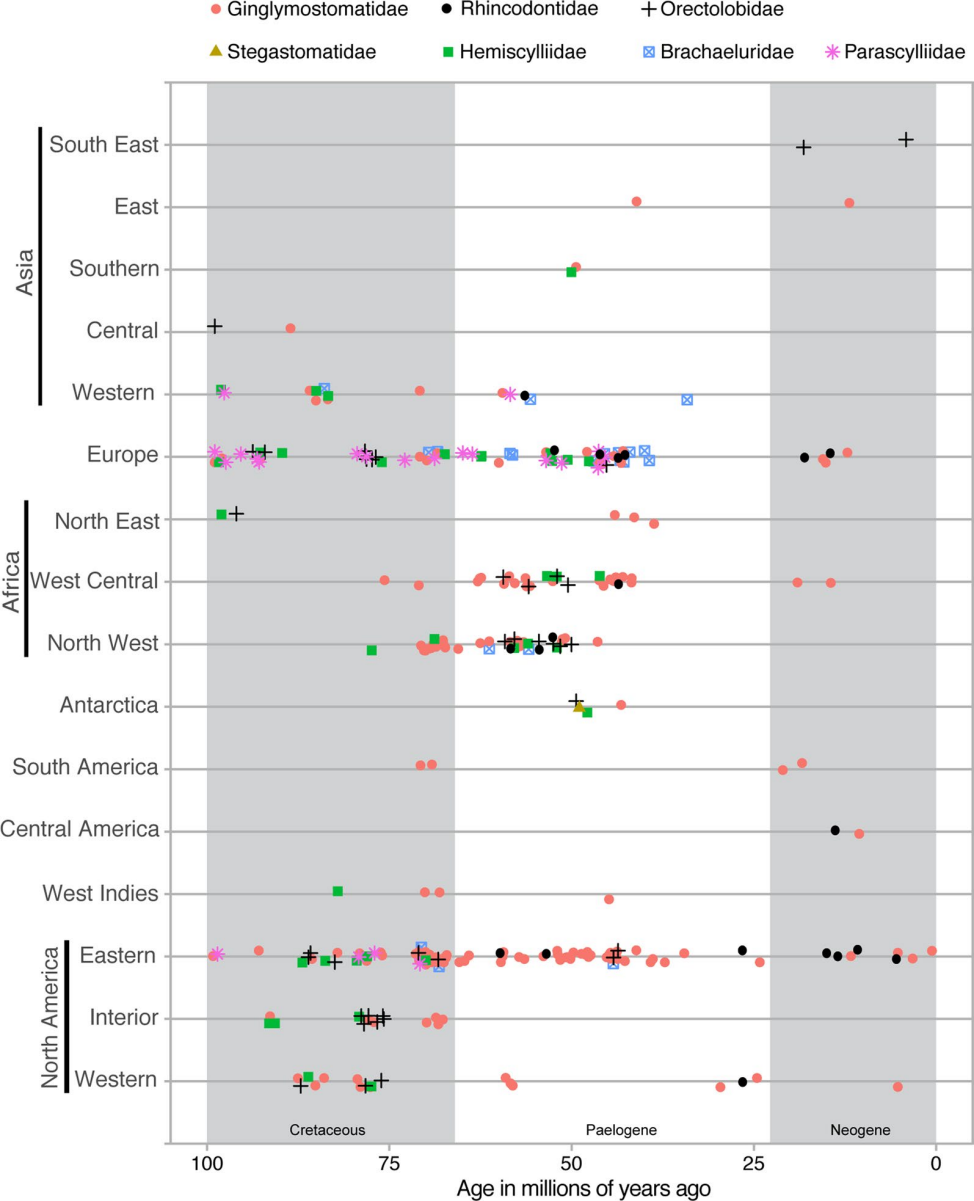
Geographical and chronological distribution of orectolobiform fossils attributed to extant families from the Late Cretaceous to recent. Fossils older than 100 MYA, fossils not attributed to a family, and suspect records (fossil identifiers 220, 273, 294) were excluded

We complied 345 fossil records, each representing a unique combination of locality, age, and species (338 published records, 7 unpublished specimens; a full account of each record and its sources are included in the supplementary data). This was an attempt at creating an exhaustive inventory of fossil carpet shark records and only duplicate records were excluded. Species attributed to multiple carpet shark families were present in the Late Cretaceous deposits of North America, Europe, and Western Asia (Fig. 1A). Fossil descriptions from Paleogene of North America were dominated reports attributed to the family Ginglymostomatidae, with fossils attributed to the Brachaeluridae and Orectolobidae from Virginia and Alabama respectively, being the two exceptions, suggesting a decline in diversity near the end of the Cretaceous period. Despite a decline in North American reports, fossils from the Paleogene of Europe were attributed to species in multiple families. We also noted reports of fossils attributed to species from multiple families from the Paleogene deposits of northwestern and west-central Africa. Fossil descriptions from central or east Asia were few and included two Late Cretaceous reports from central Asia and two Paleogene reports, one from southern Asia, and the other from east Asia. Reports of fossils attributed to family Ginglymostomatidae are common throughout the Late Cretaceous and Paleogene fossil record. With the exception of two fossils described from southeast Asia attributed to the family Orectolobidae, all fossils obtained from Neogene deposits were attributed as species belonging to the Ginglymostomatidae. Fossils attributed to the pelagic family Rhincodontidae were limited to the Paleogene or Neogene deposits, across multiple continents (Fig. 1B). Collectively, centers of carpet shark diversity appear to have shifted while members of the Ginglymostomatidae and Rhincodontidae became and remained established throughout the world.

We constructed a phylogenetic tree representing all seven carpet shark families and 13 genera. We did not capture the full species diversity within two families, Hemiscylliidae and Parascylliidae, but we did not exclude any major groups or species with an atypical biogeographical range. This tree was largely consistent with other phylogenetic results. Bootstrap support was greater than or equal to 75 % for most nodes in this tree. Nodes with lower bootstrap support included those node connecting species within families (Hemiscylliidae and Orectolobidae) and the node connecting Stegostomatidae and Rhincodontidae. Of the 345 fossils descriptions obtained for this study, 298 could readily be assigned to a species divergence with our phylogenetic tree. From each of the 298 records we attempted to build 298 time-calibrated phylogenetic trees using penalized likelihood [33, 34]. From these trees we extracted node ages for the three nodes representing divergences at different time frames (Table 1). We then determined the node age range and excluded any tree, along with its associated fossil record, that was a statistical outlier. This left us with a population of trees and their associated fossil records. Of these remaining records we selected the oldest fossil and used its minimum geological age as our calibration point for that node, leaving us with four fossil records assigned to four nodes. A final calibrated phylogenetic tree was produced using these four fossils. Mean node ages were reached in two identical runs, suggesting a stable result given our fossil data. We then examined three nodes in the tree where we had an expectation of node ages, finding that the mean node ages fit within our expectations and compared them to single point calibrations (Table 1). Node ages based on single point calibrations were consistent with our expected divergence times in two of three instances and all three predicted node ages were younger than ages obtained from multiple point calibrations. By “weeding out” uninformative or problematic fossils through a systematic process, we produced an age-calibrated phylogenetic tree of carpet sharks that was consistent with our expectation of divergence times at three different nodes. Therefore, at least with regards to carpet sharks, our method of culling and selecting fossils appears valid and provides improvement over single point calibrations.

**Table 1:** Expected and observed species divergence times in MYA

Species divergence	treePL^1^	treePL^2^	MCMC^3^	MCMC^4^	MCMC^5^	Expected	Source for expected
*G. cirratum* and *G. unami*	3.7	2.9	4.8	3.9	4	2-5	[12, 55, 56]
*O. maculatus* and *O. halei*	23.5	15.9	18.6	15.3	15.3	≤ 25	[7, 35, 36]
*P. collare* and *G. cirratum*	356.5	246.8	203.7	139.7	141.1	171–237	[14, 27, 29, 31, 57]

^1^Denotes mean age of penalized likelihood estimates using treePL before outliers were removed

^2^Denotes the same as above after outliers were removed

^3^Denotes ages inferred with four calibration points selected by our method

^4^Denotes ages inferred by using the oldest fossil previously used to calibrate the earliest split in Orectolobiformes (171 MYA)

^5^Denotes ages inferred by using the oldest fossil in our data set to calibrate the earliest split in Orectolobiformes (191 MYA)

**Fig. 2:**
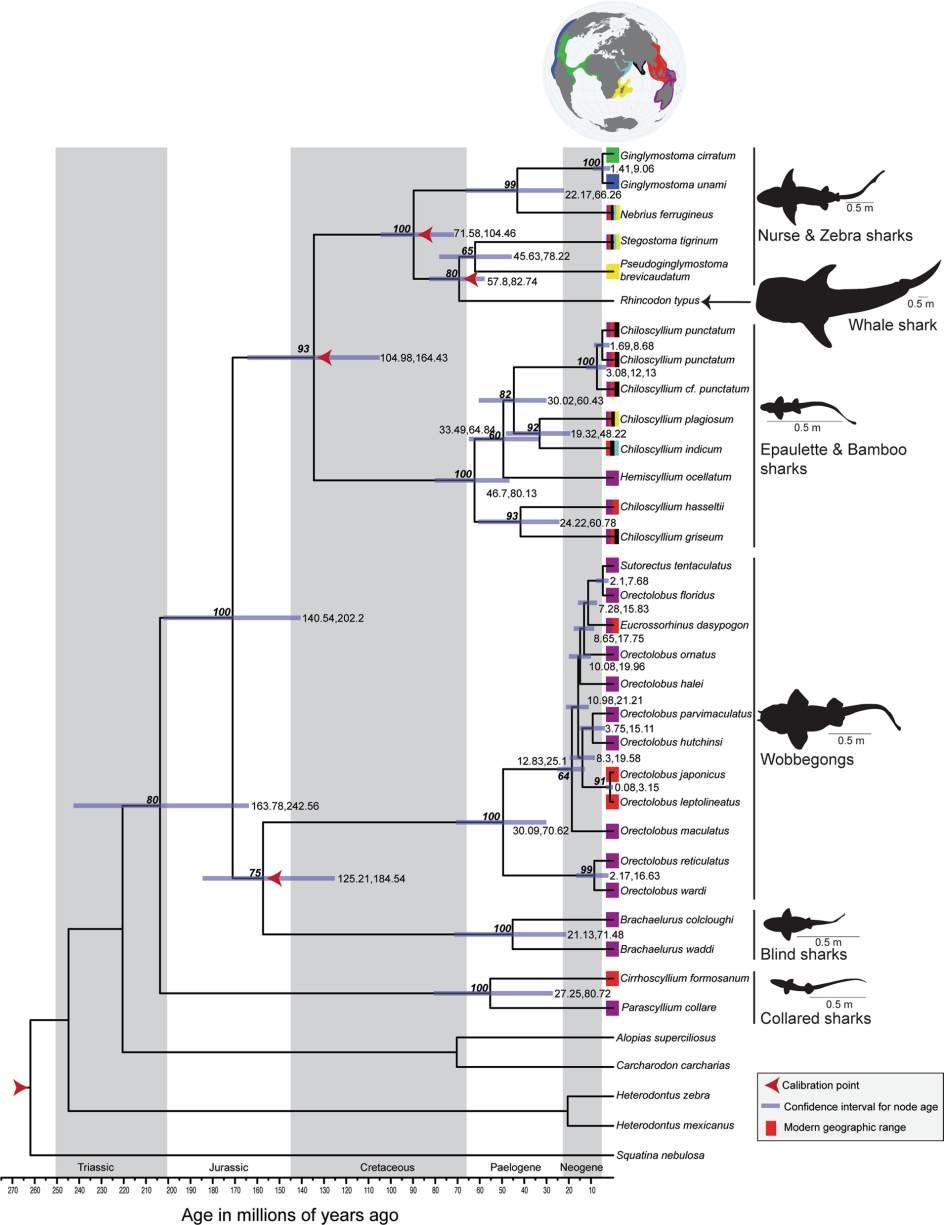
Time-calibrated phylogeny of carpet sharks based on mitochondrial DNA. Red arrows denote fossil-calibrated nodes used in the analysis. Blue bars and adjacent labels denote the confidence interval obtained using the program MCMCTree to sample the node ages. Values in italics represent bootstrap support for nodes. Colored boxes at tree tips represent modern ranges for species (blue = eastern Pacific Ocean, green = Atlantic Ocean, yellow = south western Indian Ocean, blue = north western Indian Ocean, black = central Indian Ocean, red = eastern Indian and western Pacific oceans north of Weber’s line, and Purple = eastern Indian and western Pacific oceans south of Weber’s line. Geologic periods are noted using gray and white boxes and associated text. Major clades of sharks are described by their common names and representative body form to the right of the tree. Globe created using rnaturalearth (https://github.com/ropensci/rnaturalearth)

We arrived at a Triassic or Jurassic origin of carpet sharks (Fig. 2) with a mean age of 203.7 MYA. Extant families of orectolobiform sharks diverged from one another prior to the Cretaceous–Paleogene (K–Pg) boundary, except for Rhincodontidae and Stegostomatidae, which diverged near the time of the K–Pg boundary. There was subsequent speciation following the K–Pg boundary, including a radiation event within the Orectolobidae during the Neogene.

## Discussion

Today, the greatest species richness of carpet sharks is centered within the eastern Indian and western Pacific oceans, a region that has been shaped by the collision of crustal plates during the Neogene (represented by red and purple regions in Fig. 2) [7, 35, 36]. A total of 41 species of orectolobiform sharks are present in this region including 39 species that are endemic to one or both of these oceans [10, 23, 24]. We were only able to identify a few fossils described from sites in close geographical proximity to this current center of carpet shark diversity. However, we identified locations distant to the current center of biodiversity, that contained diverse assemblages of fossil carpet shark species, representing multiple families, during the Late Cretaceous and Paleogene. Based on the data assembled here, we propose that the center of carpet shark diversity shifted to the eastern Indian and western Pacific oceans during the Cenozoic.

Fossil descriptions indicate two regions were host to diverse assemblages of carpet shark species during the Late Cretaceous. Fossil from western Eurasia suggest that elements of the Tethys seaway hosted a diverse assemblage of carpet shark species, representing multiple families [37]. Fossil evidence also suggests a diverse assemblage of carpet shark species were present in the western interior seaway and inundated Atlantic coastal plain of North America during the same time period [38–40]. Late Cretaceous shallow seas in these regions likely provided suitable habitat for carpet sharks [39, 40] and facilitated speciation. The end of the Cretaceous was marked by a decline in carpet shark diversity in North America, while diversity remained high in what is now Europe. Fossil evidence also shows a diverse assemblage of carpet shark species in west central and north Africa during the Paleogene, also likely facilitated by suitable habitat that was available at this time [41]. While fossil data is often patchy and does not fully capture the timing and extent of invasion and speciation within a region, the data clearly demonstrates that a diverse carpet shark assemblage previously occupied the Tethyan seaway and shallow seas connected to the Atlantic basin. Currently, the Atlantic basin is home to two carpet shark species, the nurse shark (*G. cirratum*) and the pelagic whale shark (*R. typus*). We conclude that the Atlantic basin was likely a cradle of carpet shark biodiversity, but that there was subsequent carpet shark extinction during the Paleogene. Collectively, the data suggests carpet sharks are capable of long-distance dispersal and colonizing nascent habitat.

The eastern Indian and western Pacific oceans have conserved carpet shark lineages, while promoting speciation within some of these lineages. Wobbegongs and blind and collared sharks are restricted to the eastern Indian and western Pacific oceans, but fossil and phylogenetic evidence suggest these groups likely evolved elsewhere [8]. Therefore, the eastern Indian and western Pacific oceans have acted as refuge following extinction of these groups in other regions. Within the eastern Indian and western Pacific oceans, the waters off Australia and New Guinea, comprising the Sahul shelf (purple region in Fig. 2), appear to have played a particularly important role in the evolution of carpet sharks [7, 8]. The waters over the Sahul shelf are host to most species of wobbegongs. Only two closely related species (*O. japonicus* and *O. leptolineatus*), along with *Eucrossorhinus dasypogon*, occur outside of Sahul shelf, to the north on the Sunda shelf [9, 42] and our phylogenetic inference suggests these species dispersed out of the Sahul shelf. Therefore, we conclude that these wobbegong species, until recently, may have been restricted to the Sahul shelf. Other carpet shark groups, including blind, epaulette (a highly specious lineage within the family Hemiscylliidae), and collared (in part) sharks are also restricted to the Sahul shelf and adjacent islands [8, 9, 15]. Therefore, we conclude that a relatively small geographical region has played a large role in conserving carpet shark diversity. Previous phylogenetic studies have also highlighted the Sahul shelf as a cradle of biodiversity [7, 8]. Within this region, both wobbegongs and epaulette sharks have speciated extensively, likely a result of allopatric speciation within the region [7, 8].

While it appears that dispersal, radiation, and extinction have shaped wobbegong, blind, and collared shark lineages, three groups, nurse, zebra, bamboo, and whale sharks, appear to defy this pattern. We noted that fossil nurse shark remains are present throughout the world during the Late Cretaceous, Paleogene, and Neogene. Furthermore, nurse shark remains have been identified after other carpet shark species disappear from the fossil record. While extant nurse shark diversity is small, the three recognized species collectively occupy a wide geographical region. Collectively, this suggests that nurse sharks acquired one or more traits that allowed them to persist in environments that led to the localized extinction of other carpet shark groups. The whale shark, and allied extinct species, shifted to planktonic feeding [13, 14]. Whale shark teeth have been found in many fossil sites including Africa, Eurasia, and the New World. The single extant whale shark species is found throughout the world [20]. The zebra shark ranges widely in the Indian Ocean, unfortunately, fossils records were few and we cannot comment at this time about its prehistoric distribution. Finally, the fossil remains of bamboo sharks have been recovered in Europe and North America. While extant species are absent from North America, the whitespotted (*C. plagiosum*) and slender (*C. indicum*) bamboo sharks are found through much of the Indian Ocean. Thus, these sharks appear to have escaped the evolutionary pressures that have shaped the remainder of carpet shark groups.

## Conclusions

The teeth of sharks, and closely related rays, are abundant in the fossil record. When used in combination with molecular phylogenetics, these fossil teeth present an opportunity to examine the prehistoric distribution of sharks and rays. We utilized this data here to explore the evolutionary and biogeographic processes that shaped carpet shark species richness. We suggest our study on carpet sharks could serve as a template for future studies utilizing the rich fossil evidence left by shark and ray species.

## Methods

### Molecular phylogenetics

Molecular [29, 43] and morphological studies [24] suggest carpet sharks form a clade within the superorder Galeomorphii. Therefore, the first step was to build a phylogenetic tree representing extant species diversity within carpet sharks. We used DNA sequence data from the mitochondrial gene NADH2, which provided us with data from all seven families and 13 genera of carpet sharks. We did not capture the full species diversity within the families Hemiscylliidae and Parascylliidae; however, we did not exclude any species having an atypical range compared to closely related species. DNA sequences were aligned as amino acid sequences using the multiple sequence aligner Muscle (v3.7; BLOSSUM62 substitution matrix) [44] and visualized in Geneious (v2019.2.1) for a manual check of alignment quality before back-translating to nucleotide. PartitionFinder2 (v2.1.1) [45] was used to identify the optimal model of sequence evolution, using Akaike information criterion modified for small sample size [46], for each codon position. Each codon position was separated into a partition and the GTR + Γ + I model was applied to each partition. Free-parameters were estimated for each codon position independently. The optimal phylogenetic arrangement was reached under a Maximum Likelihood (ML) optimality criterion using RAxML (v8.2.12) [47]. We validated our tree in two ways. First, the species arrangements in our ML tree were compared with previous findings. Second, we assessed support among 1000 bootstrap replicates. An ML tree was calculated for each bootstrap replicate under the same model of sequence evolution, again allowing free-parameters of the model to be calculated for each codon position.

### Fossil data collection and selection

We obtained fossil data by reviewing the relevant primary literature. Because some geographic regions were under-represented in our data, we made a special effort to seek out published reports from these regions. We also visited a large collection of fossil shark remains with well documented origins (G. Hubbell coll.) to supplement the published data, particularly in regards to filling-in data gaps in under-represented regions. Most fossils were described using geologic age and were converted to coarse age ranges in MYA following Walker et al. [48]. It was appropriate to use coarse age ranges as fossil teeth often aggregate in lag deposits that can prevent assignment to a narrow age range. We obtained 345 fossil records in total.

Carpet sharks have teeth that rotate to accommodate soft or hard-bodied prey [17] and the associated tooth morphology is unique among sharks [13, 18] (E. Manning pers. comm.). Within carpet sharks, the upper margin of the labial tooth face differs between families [18]. These differences facilitate the family-level classification of teeth and suggests most fossil descriptions likely represent accurate fossil classification to an extant family and not an extinct sister lineage. The loss of diagnostic tooth morphology in a clade could be problematic for incorporating fossil remains. The wobbegongs (Orectolobidae) and the whale shark (Rhincodontidae) have atypical novel tooth morphology associated with novel diets [9, 21, 49]. Wobbegong teeth superficially resemble teeth from angel sharks (Squatiniformes: Squatinidae), but characters along the upper margin can be used to assign teeth to Orectolobidae. Whale shark teeth are greatly reduced, [18] resembling teeth from other filter-feeding sharks, but root structure differs among filter-feeding shark species [13]. Given the unique morphology, we expect that teeth described in the literature were in most cases correctly identified and each fossil was assigned to a node in our tree based on its described taxonomic classification (Fig. 2). Fossils with no family level assignment reported and fossils from the genus *Pararhincodon*, which appears to represent an extinct clade, were left unassigned (47 records). Our phylogenetic reconstruction suggested the family Ginglymostomatidae is polyphyletic; therefore, fossils assigned to the genus *Pseudoginglymostoma* were grouped with fossils from the family Stegostomatidae and were considered for calibration of node 5.

We developed a bioinformatics process to rapidly evaluate each fossil record individually and identify problematic fossils. First, each fossil record was used to convert our ML tree into a chronogram based on its node assignment and age range. Because we had to repeat the process of node calibration for each of the 298 fossil records, we used a ML method described by Sanderson [33] to quickly estimate a chronogram, rather than a more computationally expensive approach. This method used an optimized ML approach to apply a relaxed molecular clock, implemented in TreePL [34]. A script was written to prepare an input file for each fossil record (298 total), with each fossil assigned to a node (nodes labeled 2–7; Table 2). TreePL was run with each input file and then node ages were collected from each output file using the R package ape (R v3.6.1; ape v5.3) [50].

**Table 2:** Nodes used to calibrate the phylogenetic tree using treePL

Node	Daughter taxa
2	*O. ornatus* and *B. warddi*
3	*G. cirratum* and *N. ferrugineus*
4	*G. cirratum* and *R. typus*
5	*S. tigrinum* and *R. typus*
6	*G. cirratum* and *H. ocellatum*
7	*C. griseum* and *H. ocellatum*

Once we generated a rate-calibrated molecular phylogeny using data from each fossil record, we needed to exclude any potentially problematic or uninformative fossil records based on the resulting time-calibrated phylogeny. To do this, we excluded any fossil that provided a node age that was outside the interquartile region at one or more of three nodes in the phylogenetic tree. These three nodes were distributed across the phylogenetic tree and where we had an expectation of node age (Table 1). We examined median node ages to determine if they were appropriate (Table 1). The remaining 110 fossils were assigned to nodes 2–6, with no remaining fossils assigned to node 7. Finally, we removed any fossils that were assigned to a node having three or fewer fossils. This resulted in removing two fossil records assigned to node 3. 108 fossil records remained.

### Fossil calibration

After we evaluated each fossil record individually and removed outliers, we identified the minimum age for the oldest fossil at each node (node 2 > 170 MYA, node 4 > 94 MYA, node 5 > 66 MYA, and node 6 > 113 MYA). Based on these four minimum calibrations of divergence times, we estimated median node ages and 95 % confidence intervals for all nodes within our phylogenetic tree using MCMCTree (v4.9j; 500,000 generations, sampling every 50 generations with a burn-in of 50,000 generations) [51]. Baseml (v4.7a) [51] was used to estimate substitution rates (0.290696) following Stein et al. [12]. Root age of our analysis was constrained to < 334 MYA, and an independent rates model was used. Mate selection, reproductive strategies, and age at maturity vary between species in this group [5, 20, 24, 52, 53]; therefore, we believed it was best to use the independent rates option available in MCMCTree. We examined results using Tracer (v.1.7.1) [54] and then replicated the analysis to check for median node age convergence.

## Data Availability

No public or private databases were used in this study to obtain fossil data; sequence data was obtained from https://www.ncbi.nlm.gov/genbank/. Fossil data was obtained from the primary literature or from accession data from the Gordon Hubbell collection with permission. Raw data is available from the figshare data repository 10.6084/m9.figshare.12595169. This repository includes all fossil records gathered from the literature and the Hubbell collection, aligned sequences, sequence partitions, ML tree with bootstrap values, individual calibrated phylogenetic trees from implementation of TreePL, summary data on node ages from each individual calibrated trees (both in total and with outliers removed), baseml control file, MCMCTree control file, MCMCTree calibration tree, calibrated phylogenetic tree based on multiple node calibration points, convergence plot for calibrated phylogenies, biogeographic model comparisons, ancestral range estimations, fossil data and node associations, node definition table, and range data for extant species.
